# Scalable hot carrier–assisted silicon photodetector array based on ultrathin gold film

**DOI:** 10.1515/nanoph-2023-0656

**Published:** 2024-01-16

**Authors:** Geunpil Kim, Hyebi Kim, Young-Uk Jeon, In Soo Kim, Soo Jin Kim, Sangsik Kim, Jongbum Kim

**Affiliations:** Nanophotonics Research Center, Korea Institute of Science and Technology (KIST), Seoul, 02792, Republic of Korea; School of Electrical Engineering, Korea University, 145, Anam-ro, Seongbuk-gu, Seoul, 02841, Republic of Korea; KIST-SKKU Carbon-Neutral Research Center, Sungkyunkwan University (SKKU), Suwon 16419, Republic of Korea; School of Electrical Engineering, Korea Advanced Institute of Science and Technology, Daejeon 34141, Republic of Korea

**Keywords:** NIR photodetection, hot carrier, extinction coefficient, gold film, photodetector array

## Abstract

Silicon (Si) offers cost-effective production and convenient on-chip integration for photodetection due to its well-established CMOS technology. However, the indirect bandgap of Si inherently limits its detection efficiency in the near-infrared (NIR) regime. Here, we propose a strategy to achieve high NIR photoresponse in Si by introducing a strong light-absorbing ultrathin gold (Au) film to generate hot carriers. Using a 4.6 nm thick-Au film deposited on Si, we achieved photoresponsivity of 1.6 mA/W at 1310 nm under zero-bias conditions, and rapid temporal responses of 7.5 and 8 μs for rise and fall times, respectively, comparable to germanium (Ge) photodiodes. By utilizing an ultrathin (<6 nm) Au film as the light-detecting layer and thicker (>100 nm) Au film as electrodes, we introduce a unique approach to design a photodiode array based on a single metal (Au) platform. Comparative analysis with a commercial beam profiler image validates the performance of our designed array. This work presents an efficient strategy for manufacturing cost-effective and scalable NIR photodetector arrays, which eliminates the need for additional insulator layers.

## 1 Introduction

Near-infrared (NIR) photodetection plays a critical role in a wide range of applications, including real-time imaging, surveillance, spectroscopy, and optical communications [1]–[5]. Many material candidates have been explored for NIR photodetectors, such as germanium (Ge) [6], III–V compound semiconductors (e.g., InGaAs) [7], [8], silicon–germanium (Si–Ge) [9], 2D materials (e.g., graphene) [10]–[12], and quantum dot (QD) [1], [13]. However, photodetectors based on Ge and InGaAs face challenges due to high fabrication costs and difficulties in on-chip integration [14]; 2D materials present difficulties in achieving large-area device fabrication; and QD-based devices suffer from issues such as high cost, demanding fabrication requirements, and low stability [15].

Silicon (Si) stands out as an attractive and cost-effective platform for photodetection owing to its abundant and low-cost fabrication techniques. Despite the wide utilization of Si photodetectors at the visible spectral regime, its detectable spectral regime is limited to wavelengths below 1100 nm due to the inherent bandgap energy of crystalline Si (1.12 eV). To achieve Si sub-bandgap detection in the NIR region, several approaches have been explored, including the introduction of bulk defect states by ion implantation, metal (Au, Ag) implantation, or laser-assisted hyperdoping [16]–[21] as well as interfacial defect states induced by film deposition on the Si surface [22], to create trap states to serve as sources of photo-excited carrier generations. However, these methods often suffer from relatively low operating speeds in the ∼ms range.

Recently, hot carrier–assisted photodetection in Schottky contact between metal and Si has emerged as a promising approach to achieving NIR photoresponse [23], [24]. Metallic nanoparticles or nanostructured surfaces have been utilized to enhance light absorption, generating hot carriers via surface plasmon resonances [25]–[27]. Despite their potential advantages such as compact integration and low-power consumption, the nanoscale patterning of metal films to employ plasmonic resonators for strong light absorption is associated with significant costs, time-consuming processes, and limited scalability. Several cost-effective methods to achieve hot carrier–assisted NIR photodiode on Si have been recently introduced; however, a comprehensive analysis of their operational speed and a practical strategy for scaling devices has not been reported yet [28], [29].

**Figure 1:**
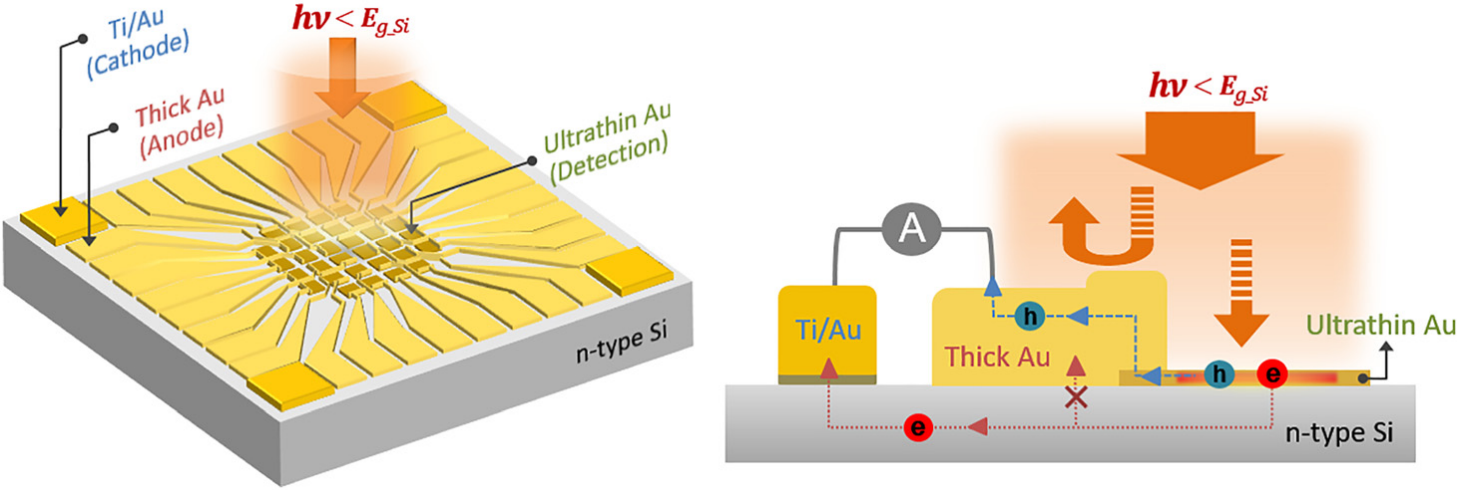
Schematic image of an ultrathin gold (Au) film–based Si photodetector array and its operating mechanism. The array is constructed with ultrathin (<6 nm) Au, thick (>100 nm) Au, and Ti/Au layers. The ultrathin and thick Au layers form a Schottky contact, while the Ti/Au layer forms an ohmic contact with n-type Si substrate. The ultrathin Au layer functions for photodetection using the hot carrier effect, and the thick Au layer serves as electrodes with no photoresponse by reflecting incoming light.

To address the limitations of nanostructure-based hot carrier photodetection, our study focuses on NIR photodetection with a patternless, lossy ultrathin gold (Au) film on an n-type Si substrate. Notably, the ultrathin Au film, a few nanometers in thickness, exhibits strong NIR light absorption due to its modified extinction coefficient, resulting in a high photoresponse of 1.6 mA/W at 1310 nm under zero-bias conditions. We determine the optimal thickness of Au film for photodetection by characterizing the thickness dependent electrical and optical properties of Au films. In addition, we quantify the frequency response and temporal response to demonstrate the impact of Au thickness on the switching speed of photodetection. We observe that the Si photodetector consisting of 4.6 nm thick-Au film exhibited fast rise/fall times of 7.5/8 μs at 1 kHz without degradation while responding to high frequencies up to 3-dB bandwidth of approximately 70 kHz. Exploiting distinct contrast in photoresponse between the ultrathin and thick Au films on Si substrate, we devise a unique method to arrange the photodetector array. As depicted in Figure 1, we designed a photodetector array, utilizing an ultrathin (<6 nm) Au film as a detection layer and a thicker (>100 nm) Au film as an electrode. The thick Au films serve solely as electrodes without any contribution to the photocurrent. Moreover, the Schottky barrier (SB) between thick Au film and Si eliminates the need for insulating materials, which are typically required in conventional detectors to electrically isolate electrodes from the substrate [10], ]. To examine the scalability and functionality of our approach, we mapped the intensity profile of the laser spot with the fabricated 32-cell array and compared it with the profile image captured by a commercial optical beam profiler. Our work demonstrates the feasibility of utilizing ultrathin Au films for a scalable, low-cost, and high-performance NIR photodetector array on a Si-based optoelectronic device.

## 2 Results and discussion

**Figure 2:**
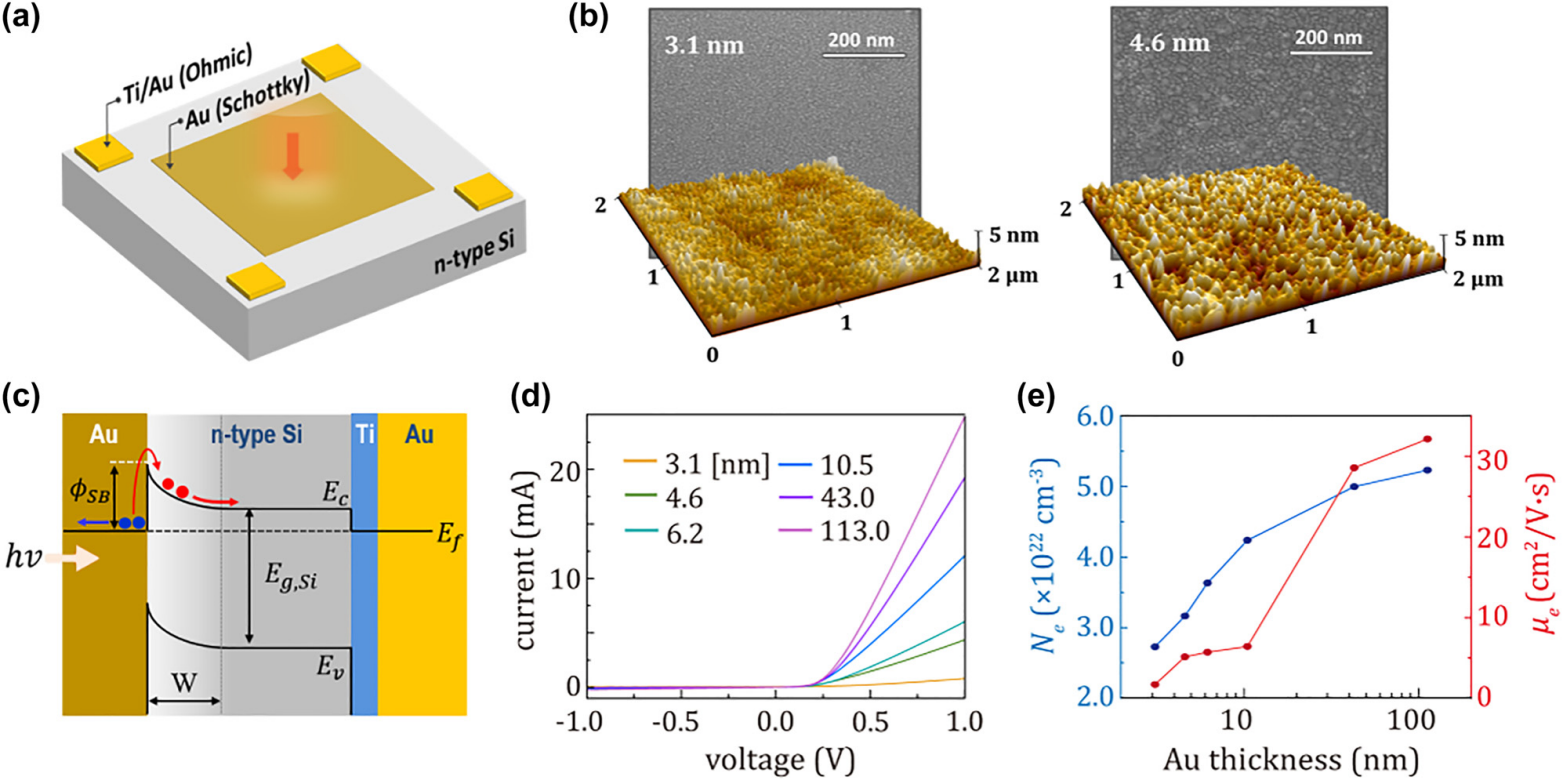
Structural and electrical characteristics of Au/Si Schottky photodiodes. (a) Schematic diagram of Au/Si Schottky photodiode. (b) Scanning electron microscope (SEM) and atomic force microscope (AFM) images of Au films with thicknesses of 3.1 nm (left) and 4.6 nm (right), respectively. (c) Energy band diagram of the Au/n-type Si Schottky junction. *E*_*c*_, *E*_*v*_, *E*_*f*_, and *E*_*g*_*Si*_ refer to the energy of the conduction band, the valence band, the Fermi level, and the Si bandgap, respectively. Blue and red circles indicate the holes and electrons, respectively. (d) Dark I–V characteristics of the photodiodes consisting of Au film with thicknesses of 3.1 nm, 4.6 nm, 6.2 nm, 10.5 nm, 43 nm, and 113 nm. (e) Carrier (electron) density (*N*_*e*_) and mobility (*μ*_*e*_) of the Au film as a function of thickness.

Figure 2a shows a schematic of our Au–Si Schottky photodiode pixel. We fabricated six different thicknesses of Au films deposited on an n-type Si substrate to identify the Au thickness to achieve optimal responsivity and switching speed. The thicknesses of Au films were set as 3.1, 4.6, 6.2, 10.5, 43.0, and 113.0 nm. A 10 nm thick-Ti and 100 nm thick-Au film were also deposited at all corners for ohmic contact with the Si substrate. The Si substrate exhibited a resistivity of 9.83 Ω cm, as characterized by Hall measurement. To check the uniformity of Au films deposited on Si substrate, we conducted a scanning electron microscopy (SEM) and atomic force microscopy (AFM), as depicted in Figure 2b. Measured images are presented only for two thinnest thicknesses of 3.1 nm and 4.6 nm. The SEM image revealed that the surface of ultrathin Au film was granular and continuous. The root-mean-squared (RMS) roughness of the Au surfaces from AFM images was found to be 0.56 nm for 3.1 nm thick-Au film and 0.93 nm for 4.6 nm thick-Au film. The RMS roughness, less than the thickness of Au films, confirmed a uniform deposition of Au films on Si without the formation of nanoisland and/or nanoparticles, even at the sub-nanometer thickness scale [33].

Figure 2c illustrates the band structure of the Au–Si Schottky junction. When incident NIR light is directed on top of the Au film (from left in the band structure illustration), hot electrons are generated at the Au film due to light absorption in the Au films. These hot electrons possess higher energy compared to thermally equilibrated carriers and, as a result, have sufficient energy to be effectively injected into the Si substrate over the SB, denoted as *ϕ*_SB_. The internal electric field in the depletion region (W) forces the injected carriers (electrons) to drift toward the cathode. Figure 2d presents the dark I–V characteristics of Au films with different thicknesses. We clearly observed that the Schottky contact was achieved between the Au films and the Si substrate regardless of the Au thickness. As the thickness of the Au film increased, the forward current at 1 V bias varied from 0.76 mA to 25 mA, indicating that thicker Au films exhibit higher conductivity. For the final step in characterizing the electrical properties of Au films, we conducted Hall measurement of Au films to monitor changes in carrier density (*N*_*e*_) and mobility (*μ*_*e*_) of Au films, as displayed in Figure 2e. We observed a gradual increase in both *N*_*e*_ and *μ*_*e*_ as the thickness of the Au film increased. We also noticed a significant change in *μ*_*e*_ occurred around a thickness of 10 nm, suggesting a huge modification in the optical properties of Au films when the Au thickness is less than 10 nm [34], [35].

**Figure 3:**
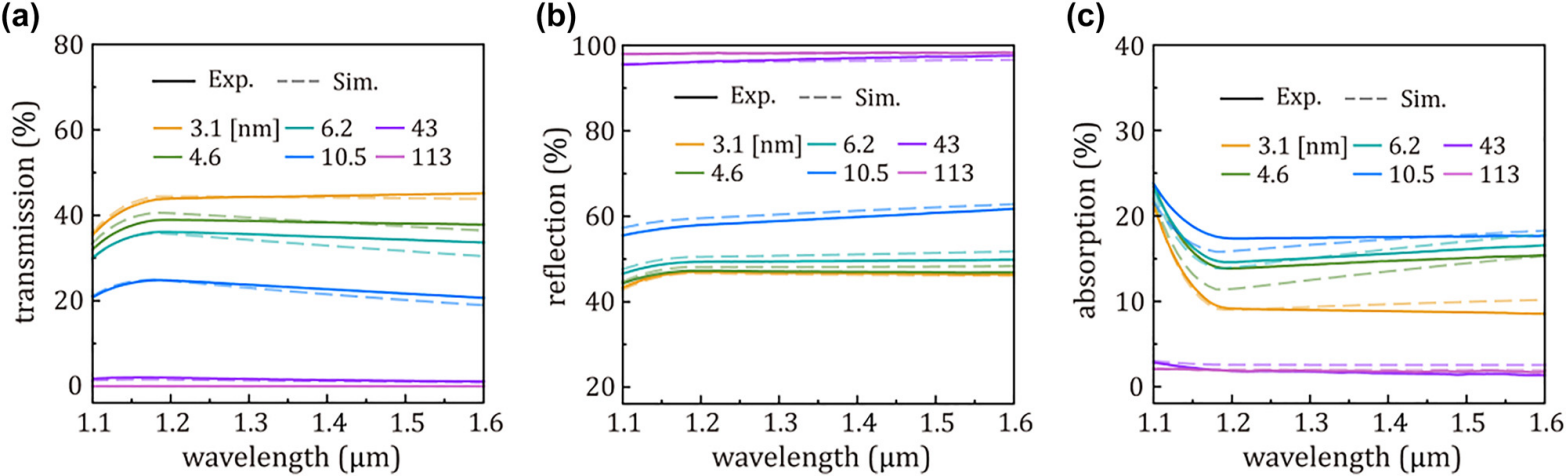
Experimental data (solid line) and simulation results (dashed line) depicting (a) transmission, (b) reflection, and (c) absorption spectra of the Au/Si photodiode structure across the spectral range from 1.1 μm to 1.6 μm. The experiment and simulation of absorption spectra are obtained from the calculation using the formula *A* = 1 − *T* − *R*.

Figure 3 shows simulated (dashed lines, Sim.) and experimentally measured (solid lines, Exp.) transmission, reflection, and absorption characteristics of the Au/Si photodiode structure with different Au thicknesses. We conducted transmission (*T*) and reflection (*R*) measurements, subsequently calculating absorption (*A*) using the formula *A* = 1 − *T* − *R*. For the numerical simulation (COMSOL), we employed the lossy Drude oscillator model to describe the optical properties of the Au films with the corresponding equation [34], ]:

$$\varepsilon \left(\omega \right)=\varepsilon_{\infty }-\frac{\omega_{p}^{2}}{\omega \left(\omega +i\Gamma_{p}\right)}$$

In this equation, *ɛ*_*∞*_ represents the high-frequency limit of permittivity while *ω*_*p*_ and Γ_*p*_ denote the plasma frequency and the damping coefficient, respectively. Given that the plasma frequency $\left(\omega_{p}=\sqrt{\frac{e^{2}N_{e}}{\varepsilon_{0}\varepsilon_{\infty }m_{e}^{*}m_{0}}}\right)$ and the damping coefficient $\left(\Gamma_{p}=\frac{\hbar e}{m_{e}^{*}m_{0}\mu_{e}}\right)$ are associated with *N*_*e*_ and *μ*_*e*_, respectively, we adjusted *ω*_*p*_ and Γ_*p*_ based on the measured *N*_*e*_ and *μ*_*e*_ for our numerical simulations. As summarized in Table 1, the *ω*_*p*_ and Γ_*p*_ of 113 nm thick Au film were assumed to be the same as those obtained from the Johnson and Christy, specially, 9.0 eV and 0.08 eV, respectively [39]. The optical properties of Au films thinner than 113 nm were scaled proportionally to the 113 nm thick-Au film. By modifying the thickness of Au films, the experimental *T* and *R* data showed good agreement with the simulation results. While the initial thickness of Au films from quartz crystal monitors (QCM) during deposition was 2.0, 3.0, 5.0, 10.0, 40.0, and 100.0 nm, the corresponding thickness based on numerical simulations was determined to be 3.1, 4.6, 6.2, 10.5, 43.0, and 113.0 nm, respectively. As the thickness of the Au film decreased, we observed an increase in transmission and a decrease in reflection. However, the trend in absorption did not align with these changes. Interestingly, we noted a maximum in absorption at Au thickness of 10.5 nm. With increasing Au thickness beyond this point, absorption rapidly diminished due to high reflection, reaching nearly negligible levels at an Au thickness of 113 nm. Considering the increase of Γ_*p*_ by a factor of 18.75 from 113 nm to 3.1 nm, sub-nanometer thick gold films exhibit sufficient lossy characteristic associated with high optical absorption, suggesting ultrathin Au films can serve as effective optical absorbers.

**Table 1:** Plasma frequency (*ω*_*p*_) and damping frequency (Γ_*p*_) of the lossy Drude oscillator model at thicknesses of 3.1 nm, 4.6 nm, 6.2 nm, 10.5 nm, 43 nm, and 113 nm used for numerical simulations.

Thickness (nm)	3.1	4.6	6.2	10.5	43	113
*ω*_*p*_ (eV)	6.5	7.0	7.5	8.1	8.8	9.0
Γ_*p*_ (eV)	1.5	0.50	0.45	0.40	0.09	0.08

**Figure 4:**
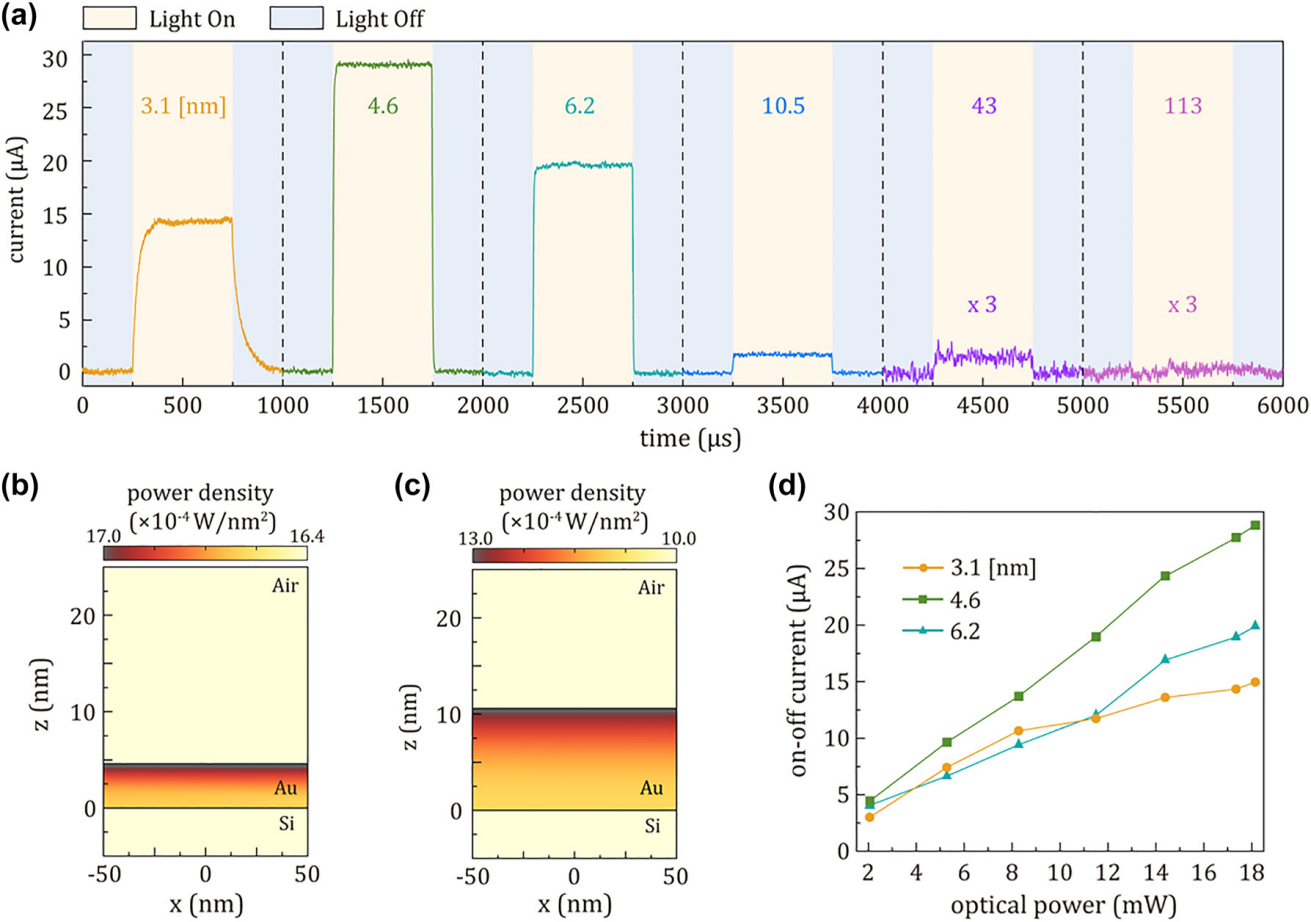
Photocurrent response and power density profiles of Au/Si Schottky photodiodes. (a) Time-resolved photocurrent (I–t) graphs of photodiodes consisting of Au film with different thicknesses ranging from 3.1 nm to 113 nm. The photocurrent was measured at a wavelength of 1310 nm with a frequency of 1 kHz and an input light intensity of 18.15 mW. The absorbed power density profile of (b) the 4.6 nm thick-Au film and (c) the 10.5 nm thick-Au film with an input laser power of 1 W at a wavelength of 1310 nm. (d) On–off current graph as a function of optical power at a wavelength of 1310 nm.

Next, we conducted the time-resolved photocurrent (I–t) measurements for all photodiodes with NIR light illumination at a wavelength of 1310 nm, as shown in Figure 4a. The incident laser, operating at a frequency of 1 kHz with a pulse width of 500 µs, has an intensity of 18.15 mW. Intriguingly, despite the 10.5 nm thick-Au film exhibiting the highest light absorption, the 4.6 nm thick-Au film showed the highest photoresponse among all samples. This observation can be attributed to both the light absorption profile and the mean-free-path (MFP) of carriers in Au films. These two parameters can define the spatial extent within which generated hot carriers can contribute to the photocurrent.

The simulated absorption profiles of the 4.6 and 10.5 nm thick-Au films are depicted in Figure 4b and c, revealing an exponential decay in light absorption from the top surface upon the NIR light illumination from the top direction. The power and the wavelength of incident light for simulation are set as 1 W and 1310 nm, respectively. Compared with two absorption profiles, we clearly observe that the light absorption in the Au film near the Schottky Junction dramatically decreases as the thickness of Au increases. Therefore, the MFP of carriers in the Au film is crucial for ensuring a high yield of photo-excited carriers to be injected into Si. Taking into account that several reported MFP values of >100 nm thick Au films range from 20 to 40 nm [34], ], we can estimate the MFP of the 4.6 and 10.5 nm thick-Au films to be approximately 3 to 6 nm and 4 to 8 nm, respectively, considering that the MFP is proportional to *μ*_*e*_. Thus, a fraction of the absorbed light within the 10.5 nm thick-Au film may not effectively contribute to photocurrent generation as it does within the 4.6 nm thick-Au film. Consequently, even a small difference in thickness can cause abrupt changes in photoresponse, particularly when the MFP approaches film thickness. Consistent with the low light absorption observed in Figure 3b, the photocurrent dramatically diminishes as the thickness of the gold films exceeds 10.5 nm, clearly shown with the 113 nm thick-Au film displaying negligible photoresponse.

Figure 4d presents the relationship between the on–off current and laser intensity for ultrathin Au films with thicknesses of 3.1 nm, 4.6 nm, and 6.2 nm. We observed that the on–off current exhibits almost a linear response with respect to the laser intensity for 4.6 and 6.2 nm thick-Au films. This linear behavior implies that the photocurrent generation increases proportionally with the increase in laser intensity. In contrast, the photocurrent of 3.1 nm thick-Au film starts to level off, preventing its linear increase. This saturation in the current-laser intensity possibly arises from the finite availability of carriers in photocurrent generations and/or the impact of trap states located at interfaces, indicating the existence of a maximum threshold for hot carrier–assisted photocurrent process in ultrathin Au film.

**Figure 5:**
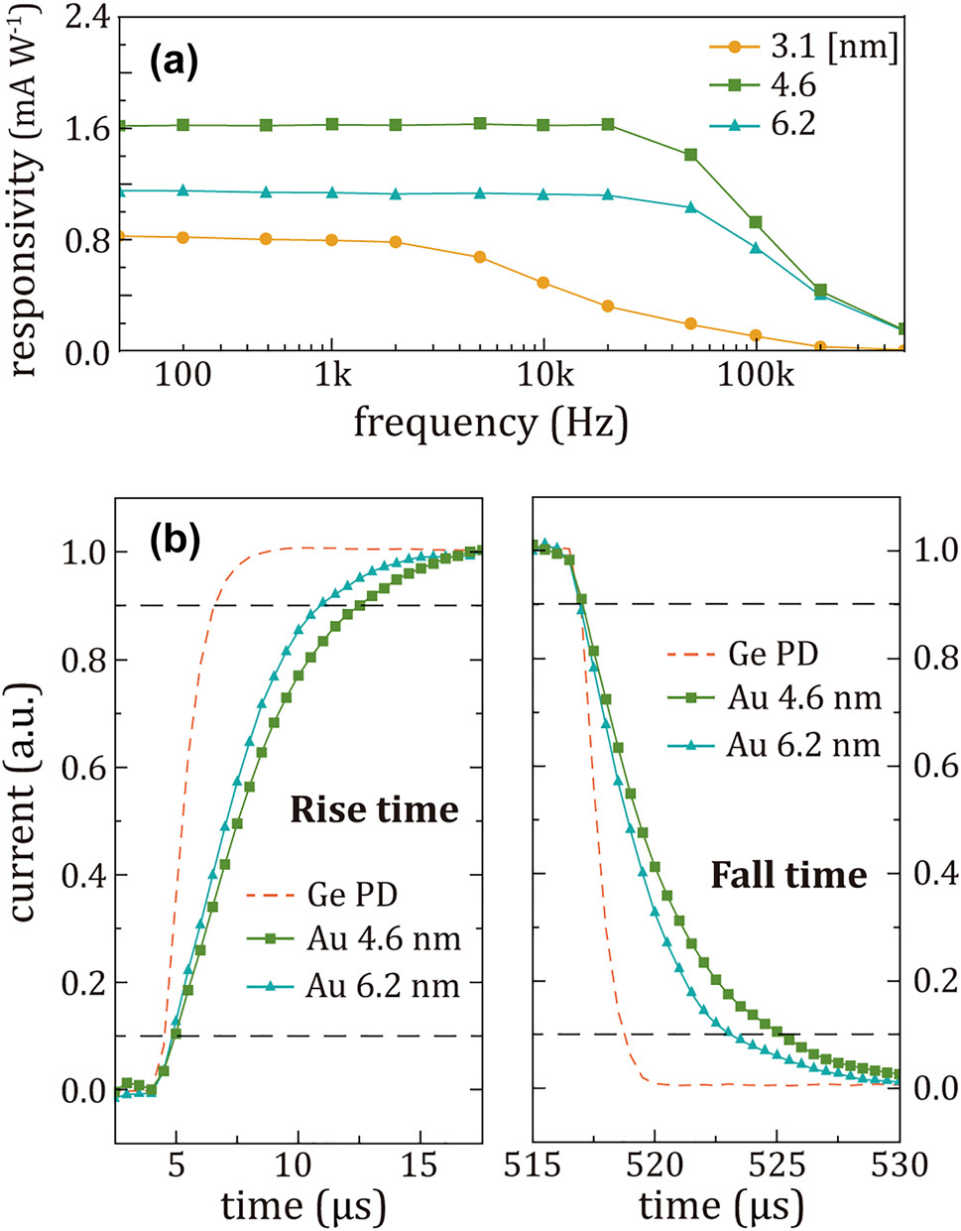
Temporal response of Au/Si Schottky photodiodes. (a) Frequency response of the photodiodes consisting of Au films with different thicknesses of 3.1 nm, 4.6 nm, and 6.2 nm under the laser illumination at the wavelength of 1310 nm. (b) Comparison of rise and fall times between the Au/Si Schottky photodiode and a commercial germanium (Ge) photodiode. The rise and fall times are calculated using the data within the black dotted lines at 0.1 and 0.9.

The switching speed of the photodetector is another critical factor in the device’s performance. Hence, we quantified the frequency response, as shown in Figure 5a. The responsivity values for the 3.1, 4.6, and 6.2 nm thick-Au photodetectors at low-frequency operation are 0.8, 1.6, and 1.2 mA/W, respectively. While both 4.6 nm and 6.2 nm thick-Au film exhibit a 3-dB bandwidth of approximately 70 kHz, the 3.1 nm thick-Au film shows a significantly lower frequency response with a 3-dB bandwidth of 8 kHz. We anticipate that the lower frequency response of 3.1 nm thick-Au film can be attributed to its higher resistance because the response speed of the photodetector is highly dependent on the resistance–capacitance (RC) time constant from an electrical perspective.

As shown in Figure 5b, we also compared the rise/fall time of our ultrathin Au–Si Schottky photodetectors with that of a commercial germanium photodiode (Ge PD, Thorlabs Inc.). The rise/fall time of the Ge PD is measured to be around 2 μs at zero bias condition, whereas our ultrathin Au–Si Schottky photodetectors exhibit rise and fall times ranging from 6 µs to 7.5 µs. Despite the lower electron mobility in ultrathin Au films compared to bulk Au, the switching performance of ultrathin Au–Si photodetector is almost on a par with a commercial Ge PD. The 6.2 nm thick-Au film exhibited improved rise/fall times at the expense of responsivity, which suggests that the thickness of Au films can be tailored depending on the requirements of their applications.

**Figure 6:**
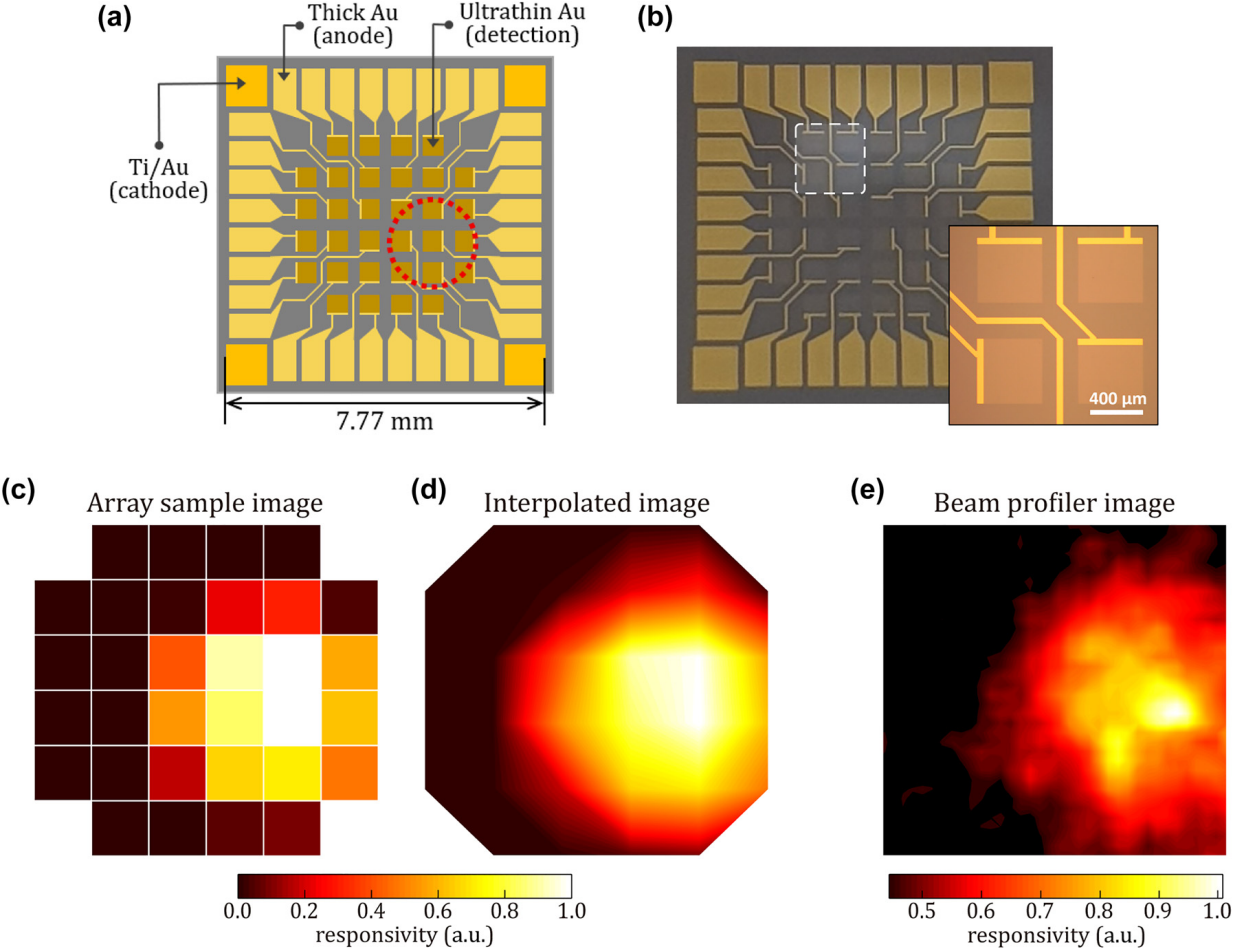
Visualization and analysis of an ultrathin gold film-based silicon photodetector array. (a) Schematic image of the fabricated ultrathin gold film–based Si photodetector array. The red dotted circle marks the laser-illuminated spot. (b) Optical microscope image of the fabricated ultrathin gold film–based Si photodetector array. The white dotted square is magnified in the shape of the “inset.” The size of each cell is 500 µm × 500 µm. (c) Individual pixel and (d) linearly interpolated beam profile image of the photodetector array with laser illumination at the red dotted circle in (a). (e) Laser intensity profile image captured from a commercial optical beam profiler (36 cells × 36 cells).

Based on the photoresponse and frequency response, we designed a photodetector array, as illustrated in Figure 6a. The ultrathin Au film (<6 nm) is suited for efficient NIR photodetection, while the thick gold film (>100 nm) is ideal for serving as the anode layer due to its excellent conductivity without any photoresponse. Remarkably, the device architecture does not require an insulator, the fabrication process can be simplified, allowing us to create a compact photodetector array that offers scalability and flexibility in layout design. For the fabrication of a photodiode array, 4.6 nm thick-Au film was deposited on the Si substrate, and a 32-cell array was patterned by a wet etching process with gold etchant. The size of each cell was set to 500 μm × 500 μm. Then, a 100 nm thick Au film was patterned to define the anode electrode. To ensure a robust connection between the detecting area and the electrode, a 50 μm × 500 μm area was strategically overlapped. The same patterning process was carried out for cathode contacts with the Ti/Au film. Therefore, the photodetector array can be constructed using a single metal (Au) platform by selectively varying its thickness and optimizing contact properties with Si.

Figure 6b shows an optical image captured with an optical microscope of the fabricated photodiode array comprising 32 cells. The area defined by white dashed lines is enlarged to provide better visual inspection (or image) of the device; in the magnified image, the well-connected interface between the thick and ultrathin Au films is displayed. The red dotted circle in Figure 6a indicates the spot where the photodetector array is illuminated by the laser beam. Figure 6c and d depict the experimentally measured responsivity images of the 32-cell photodiode array: (c) as is individually and (d) interpolated responsivity images. For the interpolation, we performed the bilinear interpolation using OriginPro 9.0 (OriginLab Corp). For comparison, we also measured the intensity profile of the laser spot with a commercial optical beam profiler (DataRay Inc.), as shown in Figure 6e. Notably, the beam profile obtained from the fabricated array is in excellent agreement with the commercial beam profiler image, validating the reliability and the performance of our photodetector array. This result demonstrates the scalability of our ultrathin Au–Si photodetector array, facilitated by its simple and insulator-free design.

## 3 Conclusions

In summary, we have explored the hot carrier–assisted NIR photoresponse in Au/Si Schottky junctions by precisely tuning the thickness of Au films. The 4.6 nm thick-Au film deposited on n-type Si exhibited excellent photoresponsivity, reaching 1.6 mA/W at a wavelength of 1310 nm under zero bias, with rapid rise/fall times of 7.5/8.0 μs at 1 kHz. Both the 4.6 nm and 6.2 nm thick-Au films revealed a 3-dB bandwidth of approximately 70 kHz, providing their potential for high-speed optoelectronic devices. Ultrathin Au films (with thicknesses less than 6 nm) exhibit high absorption near the Au/Si interface, leading to efficient hot carrier generation and carrier injections into Si. In contrast, thicker Au films (>100 nm) within the same configuration functioned effectively as electrodes due to the absence of photocurrent and high conductivity for charge extraction. The photodetector array, featuring ultrathin Au film for NIR detection and thick Au film as electrodes, was able to replicate the results of a commercial beam profiler. Our Au/Si photodetector configuration can be readily scaled further, offering a cost-effective solution to a wide range of applications.

## 4 Experimental section

### 4.1 Sample preparation

A monocrystalline n-type (100) silicon substrate, polished on both sides, with a resistivity of 1–10 Ω cm and a thickness of 500 μm, was selected for the experiment. Each sample was thoroughly cleaned with acetone and methanol, and the native oxide layer was effectively removed by a buffered oxide etcher (BOE) solution for a duration of 15 s. To fabricate a singular Au film photodiode, an Au film was initially deposited on a 0.5 cm × 0.5 cm area at the center of the Si substrate through e-beam evaporation to define the detection region. Subsequently, 10 nm thick Ti and 100 nm thick Au were deposited at the corner of the substrate through e-beam evaporation to construct the cathode of the device. For Hall measurement, we separately prepared Si substrate deposited Au film with various thicknesses. The Au film was deposited on entire area (1 cm × 1 cm) of Si substrate, and 10 nm thick Ti and 100 nm thick Au film additionally deposited on Au film at the corner of the substrate to achieve stable electrical contact of Au film.

### 4.2 Sample characterization

Surface imaging was conducted using a field-emission scanning electron microscope (FE-SEM, Nova-SEM) and atomic force microscopy (AFM, Park XE-100), with supplementary details found in the corresponding section. For electrical characterization, a semiconductor parameter analyzer (Keithley 4200) was used to measure current–voltage (I–V) characteristics, while the Ultra-Fast I–V module (Keithley 4225-PMU) was employed for current–time (I–t) characteristics measurements. All evaluations were conducted at room temperature. Optical power monitoring was executed using a hand-held optical power meter (Newport Model 843-R-USB) and a wand-style photodiode power sensor (Newport Model 818-ST2-IR/DB). Comparative temporal response analysis was achieved using a commercial germanium photodiode, specifically the Thorlabs Model FDG03. The optical beam profiler used for comparison with our photodetector array is the DataRay Model TaperCamD20-15-UCD23. Transmittance and reflection data were collected using a UV-visible spectrophotometer (SHIMADZU UV-3600 plus), spanning wavelengths from 1100 to 1600 nm. Subsequently, absorption was calculated based on the transmission and reflection data.
